# Influence of VEGF-A, VEGFR-1-3, and neuropilin 1-2 on progression-free: and overall survival in WHO grade II and III meningioma patients

**DOI:** 10.1007/s10735-020-09940-2

**Published:** 2021-02-02

**Authors:** Simon Bernatz, Daniel Monden, Florian Gessler, Tijana Radic, Elke Hattingen, Christian Senft, Volker Seifert, Michael W. Ronellenfitsch, Karl H. Plate, Patrick N. Harter, Peter Baumgarten

**Affiliations:** 1 Neurological Institute (Edinger Institute), University Hospital Frankfurt, Goethe-University, Frankfurt, Germany; 2 Department of Neurosurgery, University Hospital Frankfurt, Goethe University, Schleusenweg 2-16, 60528 Frankfurt am Main, Germany; 3 Institute of Clinical Neuroanatomy, Goethe-University, Frankfurt, Germany; 4 Department of Neuroradiology, University Hospital Frankfurt, Goethe-University, Frankfurt, Germany; 5 Department of Neuro-Oncology, University Hospital Frankfurt, Goethe-University, Frankfurt, Germany; 6 Frankfurt Cancer Institute (FCI), Goethe-University, Frankfurt, Germany; 7 German Cancer Consortium (DKTK), Partner site Frankfurt/Mainz, Germany; 8 German Cancer Research Center (DKFZ), Heidelberg, Germany

**Keywords:** Meningioma, VEGF, VEGF receptors, Neuropilin, Malignant meningioma

## Abstract

Higher grade meningiomas tend to recur. We aimed to evaluate protein levels of vascular endothelial growth factor (VEGF)-A with the VEGF-receptors 1-3 and the co-receptors Neuropilin (NRP)-1 and -2 in WHO grade II and III meningiomas to elucidate the rationale for targeted treatments. We investigated 232 specimens of 147 patients suffering from cranial meningioma, including recurrent tumors. Immunohistochemistry for VEGF-A, VEGFR-1-3, and NRP-1/-2 was performed on tissue micro arrays. We applied a semiquantitative score (staining intensity x frequency). VEGF-A, VEGFR-1-3, and NRP-1 were heterogeneously expressed. NRP-2 was mainly absent. We demonstrated a significant increase of VEGF-A levels on tumor cells in WHO grade III meningiomas (p = 0.0098). We found a positive correlation between expression levels of VEGF-A and VEGFR-1 on tumor cells and vessels (p < 0.0001). In addition, there was a positive correlation of VEGF-A and VEGFR-3 expression on tumor vessels (p = 0.0034). VEGFR-2 expression was positively associated with progression-free survival (p = 0.0340). VEGF-A on tumor cells was negatively correlated with overall survival (p = 0.0084). The VEGF-A-driven system of tumor angiogenesis might still present a suitable target for adjuvant therapy in malignant meningioma disease. However, its role in malignant tumor progression may not be as crucial as expected. The value of comprehensive testing of the ligand and all receptors prior to administration of anti-angiogenic therapy needs to be evaluated in clinical trials.

## Introduction

Meningiomas are the most common brain tumors, mostly of benign nature (Louis et al. 2016). However, WHO grade II and III tumors tend to recur and malignant progression is observed in some meningiomas in the course of the disease. In our cohort, we previously reported a 70% recurrence rate within 5 years (Baumgarten et al. 2016b). New methylation-based classifications claim to be even more precise than the WHO classification system (Sahm et al. 2017; Nassiri et al. 2019) but this analysis is only available for a very limited number of centers. Angiogenesis affects glial brain tumors (Plate et al. 1992; Baumgarten et al. 2016a) but the comprehensive impact is still not clear in higher grade meningiomas. Our previous study with a limited amount of patients and a short follow-up investigating vascular endothelial growth factor (VEGF) and its two receptors, vascular endothelial growth factor receptor (VEGFR)1 (flt-1) and VEGFR-2 (flt-1/KDR), demonstrated heterogeneous expression levels but no influence on progression-free survival (PFS) or overall survival (OAS) in WHO grade II and III meningiomas (Baumgarten et al. 2013). In contrast, a recent study of a small cohort showed an upregulation of VEGF in higher WHO grades (Reszec et al. 2015). The major co-receptor of VEGFR-1 and VEGFR-2, Neuropilin-1 (NRP-1), has only been investigated in angiomatous meningiomas on an mRNA level so far (Nassehi et al. 2013) while there is a lack of data on higher grade meningiomas. No data are available for VEGFR-3 (flt4) which was demonstrated to be upregulated in VEGF-A driven angiogenesis, at least in gliomas in vitro and in vivo (Shibuya and Claesson-Welsh 2006). To our knowledge, no data about the co-receptor Neuropilin-2 (NRP-2) in meningiomas are available to date. NRP-1 and NRP-2 promote the binding of VEGF to its receptors (Soker et al. 1998; Kawasaki et al. 1999; Pan et al. 2007). Clinical phase II studies using the anti-VEGF antibody Bevacizumab in recurrent malignant meningiomas failed to show improved OAS, but Bevacizumab could at least stabilize the disease in 88% of the patients (Shih et al. 2016). This is in line with the findings of a retrospective case series (Nayak et al. 2012; Lou et al. 2012). Sunitinib is a small molecule tyrosine kinase inhibitor that directly targets VEGFR-2, among others, and has already been tested in pretreated higher grade meningiomas (Kaley et al. 2015). In this study, higher VEGFR-2 expression was beneficial for PFS. However, prospective randomized trials are missing.

The aim of our study was to comprehensively analyze and evaluate protein expression levels of the VEGF-A-driven system with VEGFR-1-3 as well as the co-receptors NRP-1 and -2 in WHO grade II and III meningiomas in order to elucidate the rationale for adjuvant treatment targets.

## Materials and methods

### Patient material

We investigated 232 specimens (including 37 repetitive cores that were excluded in statistical analyses) of 147 patients who suffered from cranial meningioma and underwent surgical resection in our department between September 2000 and December 2014. Patients with recurrent tumors as well as patients undergoing primary surgical treatment for meningioma were included in the study. Tumor tissue of a first recurrence was available in 28 patients, a second recurrence in 11 patients, and a third recurrence in 5 patients. Representative cores of all specimens were transferred to tissue micro arrays (TMA) for immunohistochemistry with inclusion of repetitive specimens. The diagnosis was re-evaluated and confirmed by at least two neuropathologists (PB and PNH) using clinical routine HE-staining and immunohistochemistry (IHC) for the Ki67-antigen and the epithelial membrane antigen (EMA) following standard routine protocols. Histological brain invasion was re-evaluated in order to determine whether central nervous system tissue was present in the specimens which was the case in 177/232 specimens. The Ki67-antigen is only mentioned as an additional diagnostic tool in this study since it has been previously published that Ki67-expression in this cohort influences progression-free survival independently (Baumgarten et al. 2016b). The Simpson score was retrieved from surgical reports if it was given in the report which was the case in 130/232 specimens. WHO grades were evaluated according to the 2016 WHO guidelines (Louis et al. 2016).

### Immunohistochemistry

Tumor sections (3 µm) were used for immunohistochemistry for VEGF-A, VEGFR-1-3, NRP-1/-2, and Ki67. Tissue labeling for all antigens was performed using the DiscoveryXT immunohistochemistry system (Ventana, Strasbourg, France) with standardized protocols as published before (Baumgarten et al. 2014, 2015). The following antibodies and dilutions were used: mouse IgG2b anti-human VEGF, dilution 1:100 (clone: MAB293; R&D Systems, Minneapolis, MN, US), mouse IgG1 anti-human VEGFR-1, dilution 1:50 (clone: ab9540; Abcam, Cambridge, UK), monoclonal rabbit anti-human VEGFR-2, dilution 1:100 (clone: 55B11; Cell Signalling, Danvers, MA, US), goat IgG anti-human VEGFR-3, dilution 1:500 (clone: AF349; R&D Systems, Minneapolis, MN, US), monoclonal rabbit anti-human NRP-1, dilution 1:100 (clone: ab81321; Abcam, Cambridge, UK), and goat IgG anti-human NRP-2, dilution 1:50 (clone: AF2215; R&D Systems, Minneapolis, MN, US). Glioblastoma samples with a high expression of each factor served as positive controls.

### In situ hybridization

In situ hybridization (ISH) for the soluble ligand VEGF-A has been performed in our previous study on meningiomas in which a steady overlap with the same anti-VEGF-A antibody could be demonstrated (Baumgarten et al. 2013). The same has been done in glioblastomas (Baumgarten et al. 2016a). Therefore, we did not repeat ISH for VEGF-A in the present study.

### Scoring of protein levels on immunohistochemically stained specimens

For evaluation of IHC, we used a validated histological scoring system multiplying staining intensity (0–3 points) with the frequency of stained cells or vessels of each represented intensity. All products were then added together to yield a specific score between 0 and 300 points in a semi-quantitative scoring system (Histo-score) (van Netten et al. 1987; Kirkegaard et al. 2006). For example, in a case with 50% negative cells, 30% with low intensity (1), 10 % with moderate (2), 10% with strong (3) staining, the score would be calculated as follows: 50 × 0 + 30 × 1 + 10 × 2 + 10 × 3 = 80 points. Every specimen was scored by two independent investigators and averaged. If the inter-reader difference was greater than 25%, the cases were reviewed a second time by both readers simultaneously and a common agreement was found. VEGF-A expression was scored separately on tumor cells and on tumor vessels.

### Statistical analyses

Statistical analysis and Figure design were performed using the JMP 14.0 software (SAS, Cary, NC, USA), GraphPad Prism 6 (GraphPad Software Inc., La Jolla, USA), and Gimp2. Evaluation of the IHC preparations and photographic documentation was performed using an Olympus BX50 light microscope. A significance level of alpha = 0.05 was chosen for all tests (p = 0.05-0.01 → *; p < 0.01–0.001 → **; p < 0.001→ ***). Survival analyses were performed using Kaplan–Meier analyses, defining the date of surgery as the starting point. For survival analyses, we performed a median split in primary tumors. High levels were defined as ≥ median. In cases where the median was 0, all values higher than 0 were defined as “high”. To compare protein levels, we applied the unpaired student’s *t* test and the *F* test. Bivariate analysis was used to determine the correlation between ligand and receptor levels. In cases of multiple testing, the Bonferroni adjustment was done by applying the Dunn method. In order to compare the Kaplan–Meier survival curves, we used the Log-rank test for censored data. Proportional hazard ratio was performed for progression-free survival to identify independent influencing factors. Due to the lack of univariate factors, proportional hazard ratio was not performed for overall survival.

## Results

### Patient cohort

Patient data including gender, age, Karnofsky performance scale (KPS), Simpson score, and number of primary and recurrent cases are summarized in Table 1.

**Table 1:** Summary of patient data

	WHO grade II	WHO grade III	Total
Male	56	7	63
Female	80	4	84
Median age (range)	58 (17–84)	56 (30–78)	57 (17–84)
KPS preoperative	90 (20–100)	80 (60–100)	90 (20–100)
KPS postoperative	90 (20–100)	70 (30–90)	90 (20–100)
Simpson I	46	3	49
Simpson II	39	2	41
Simpson III	13	0	13
Simpson IV	24	3	27
Primary meningioma	125	8	133
1st Recurrence	24	4	28
2nd Recurrence	7	4	11
3rd Recurrence	2	3	5

*KPS* Karnofsky performance scale (KPS)

The table summarizes the patient characteristics of the investigated cohort

### Protein levels and WHO grade

**Fig. 1:**
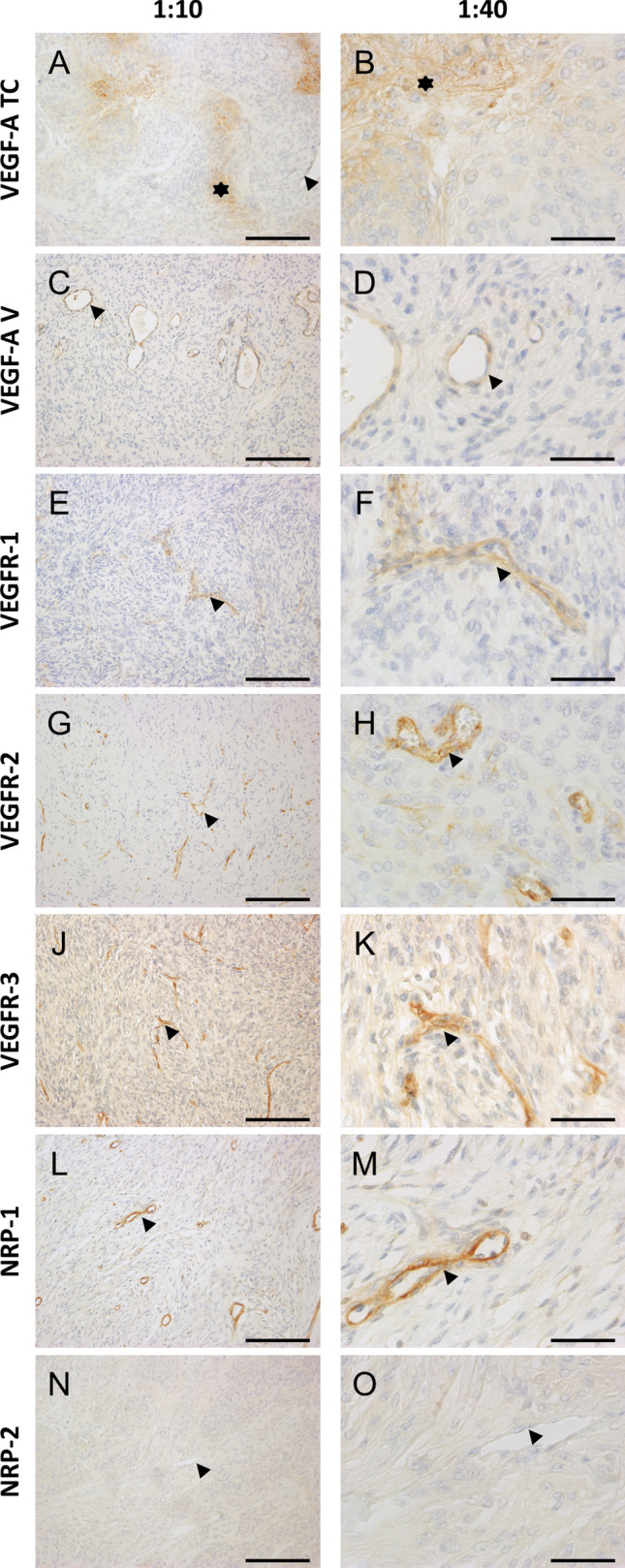
Immunohistochemistry of all factors in WHO grade II tumors. Magnification: first column 1:10, scale bar 1000 µm, second column 1:40, scale bar 200 µm. **a**/**b** VEGF-A on tumor cells (asterisk) and tumor vessels (arrowhead). **c**/**d** vessel-dominant VEGF-A expression (arrowheads). E/F) VEGFR-1 on tumor vessels (arrowheads). **g**/**h** VEGFR-2 on tumor vessels (arrowheads indicating a vessel in G and an endothelial cell in H). **j**/**k** VEGFR-3 on tumor vessels (arrowheads). L/M) NRP-1 on tumor vessels (arrowheads). N/O) NRP-2 negative tumor vessels (arrowheads). Abbreviations: vascular endothelial growth factor (VEGF), VEGF-receptor (VEGFR), neuropilin (NRP)

**Fig. 2:**
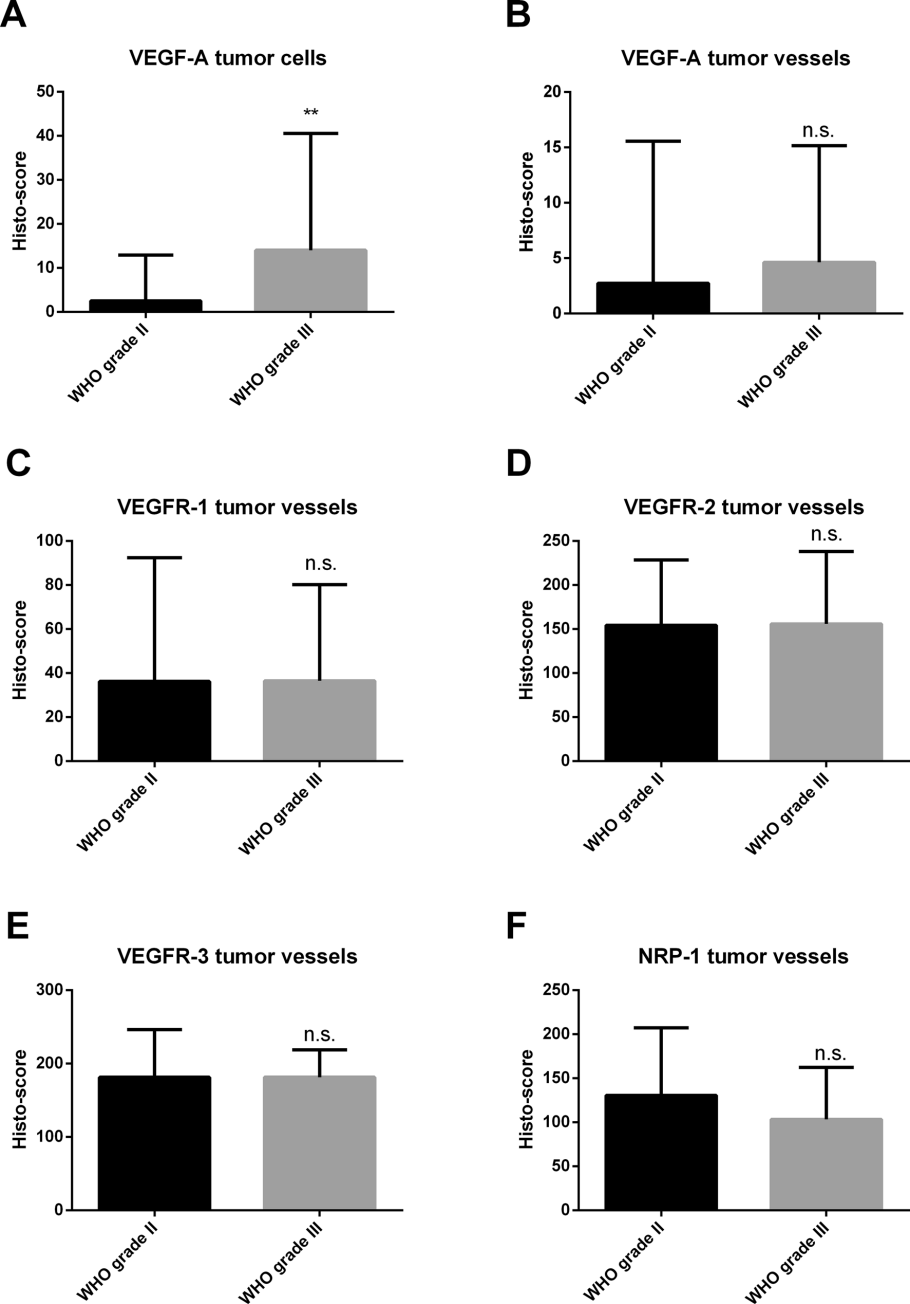
Comparison of protein expression between WHO grades II and III in primary meningiomas. Significant difference for VEGF-A expression on tumor cells (unpaired *t* test, p = 0.0098). Boxplots with error bars showing the range. Abbreviations: vascular endothelial growth factor (VEGF), VEGF-receptor (VEGFR), neuropilin (NRP), not significant (ns)

In patients undergoing primary surgery, VEGF-A and its receptors VEGFR-1, VEGFR-2, VEGFR-3, and NRP1 were expressed in a heterogeneous pattern (Fig. 1). VEGF-A on tumor cells and VEGF-A on tumor vessels were expressed in a different pattern: tumors with high levels on tumor cells did not always show high vascular levels in the same region and vice versa (Fig. 1a–d). NRP-2 was not expressed in any of our primary tumors (Fig. 1n–o). NRP-2 was consequently excluded from further analyses. In patients undergoing primary surgery, we identified a significant increase of VEGF-A on tumor cells in WHO grade III meningiomas (mean ± SD or SEM) compared to WHO grade II meningiomas (unpaired *t* test: p = 0.0098, difference between means 11.48 ± 4.372, 95% confidence interval 2.822 to 20.13, Fig. 2a). Interestingly, the expression of VEGF-A on tumor vessels did not differ between WHO grades II and III (Fig. 2b). Furthermore, there was no significant difference in protein levels for VEGFR-1-3 and for the co-receptor NRP-1 between WHO grades II and III (Fig. 2c–f).

### Differences in recurrent disease

**Fig. 3:**
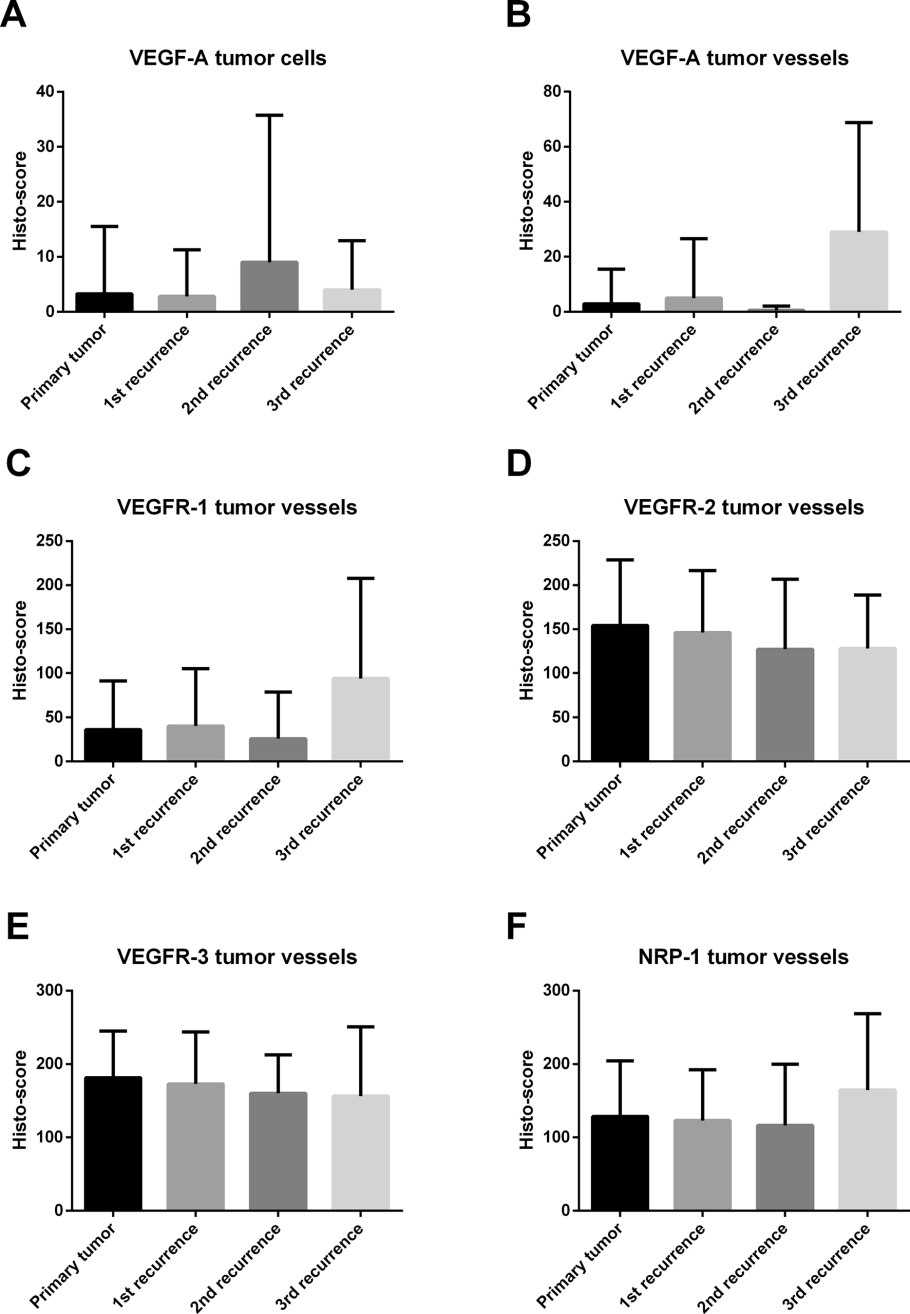
Comparison of protein expression between primary tumor and first, second, and third recurrence. Box-Whisker-plots are depicted. Abbreviations: vascular endothelial growth factor (VEGF), VEGF-receptor (VEGFR), neuropilin (NRP)

Comparing primary and recurrent tumors, there was a trend towards an increase of VEGF-A expression on tumor vessels in the third recurrence as compared to the primary situation (Fig. 3b), however significance was lost after Bonferroni correction A trend towards an increase was also observed for the receptor VEGFR-1 in the third recurrence compared to the primary situation (Fig. 3c), but once again significance was lost after the Bonferroni correction for multiple testing was applied. All other investigated factors did not show significant differences comparing primary tumors with first, second, or third recurrence with and without Bonferroni correction (Fig. 3).

### Correlation of ligand and receptor levels

Correlation analysis of protein levels of VEGF-A with the receptors VEGFR-1 -3 and with the co-receptor NRP-1 showed a significant correlation between VEGF-A and VEGFR-1 on tumor cells and tumor vessels (p < 0.0001). VEGFR-3 was significantly correlated with VEGF-A on tumor vessels (p = 0.0034) but not tumor cells. All correlations are summarized in Table 2.

**Table 2:** Correlation of ligand with receptor levels

Receptor	Location VEGF-A	Probability > [p]
VEGFR-1	Tumor cells	**< 0.0001**
VEGFR-1	Tumor vessels	**< 0.0001**
VEGFR-2	Tumor cells	0.6697
VEGFR-2	Tumor vessels	0.1813
VEGFR-3	Tumor cells	0.3375
VEGFR-3	Tumor vessels	**0.0034**
NRP-1	Tumor cells	0.5138
NRP-1	Tumor vessels	0.0546

*VEGF* vascular endothelial growth factor (VEGF), *VEGFR* VEGF-receptor (VEGFR), *NRP* neuropilin (NRP)

The table shows the results of the bivariate fit of receptor levels by ligand levels for VEGF-A on tumor cells and tumor vessels

### Impact on progression-free survival

**Fig. 4:**
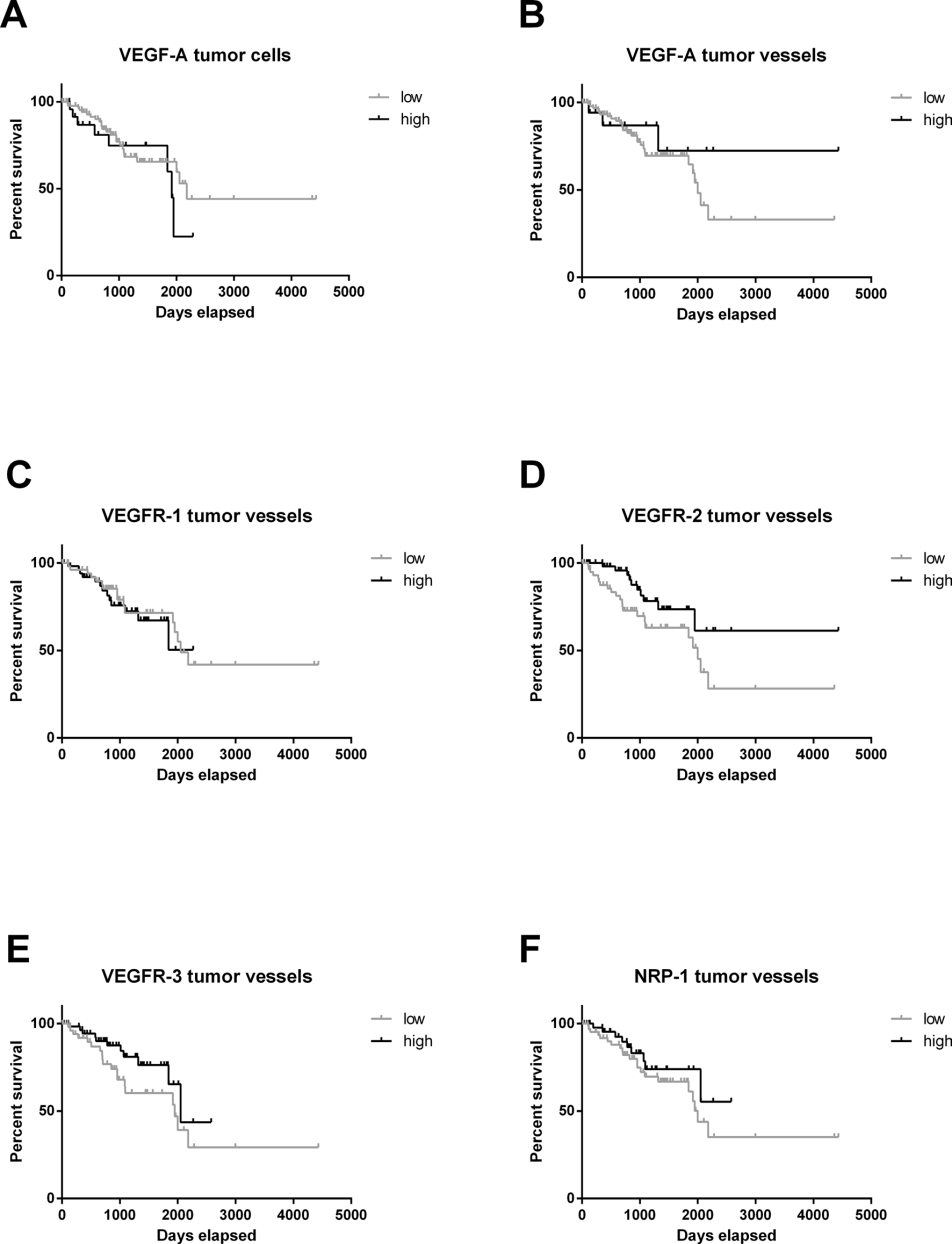
Kaplan Meier curves comparing progression-free survival of patients with high or low protein expression. Patients were stratified by the median. No significant difference was shown for (**a**) VEGF-A on tumor cells, (**b**) VEGF-A on tumor vessels and (**c**) VEGFR-1 on tumor vessels. VEGFR-2 was positively associated with patient PFS (Log-rank, p = 0.0340; Wilcoxon, p = 0.0175). No significant difference was shown for (**e**) VEGFR-3 and (**f**) NRP-1. Abbreviations: vascular endothelial growth factor (VEGF), VEGF-receptor (VEGFR), neuropilin (NRP)

Using median split data analysis, we found that the expression of the key ligand VEGF-A on tumor cells (Fig. 4a) or on vessels (Fig. 4b) and most of the further examined receptors and co-receptors (Fig. 4c–f) did not impact PFS. (Figure 4). Interestingly, the most important receptor for VEGF-A, VEGFR-2, was positively associated with patient PFS (median survival was 2000 days (5.5 years) for low levels and undefined for high levels, Log-rank, p = 0.0340; 95% confidence interval of ratio: 1.062 to 4.247 and 0.2355 to 0.9416; Fig. 4d). Multivariate analyses including the Ki67-proliferation index, the Simpson score and VEGFR-2 expression did not confirm VEGFR-2 as an independent factor for PFS but only Ki-67 proliferation index (Table 3).

**Table 3:** Multivariate analysis for progression free survival

Factors	No. of parameters	Probability > Chi^2^
Ki67 ≥ 5%	1	**0.0162**
Simpson score	3	0.1914
VEGFR-2	1	0.0986

*VEGFR-2* vascular endothelial growth factor receptor 2 (VEGFR-2), *no* number (no.)

The table shows the results of the proportional hazard ratio with only the Ki67 proliferation index of 5% or more being an independent factor for progression-free survival

### Impact of protein levels on overall survival

**Fig. 5:**
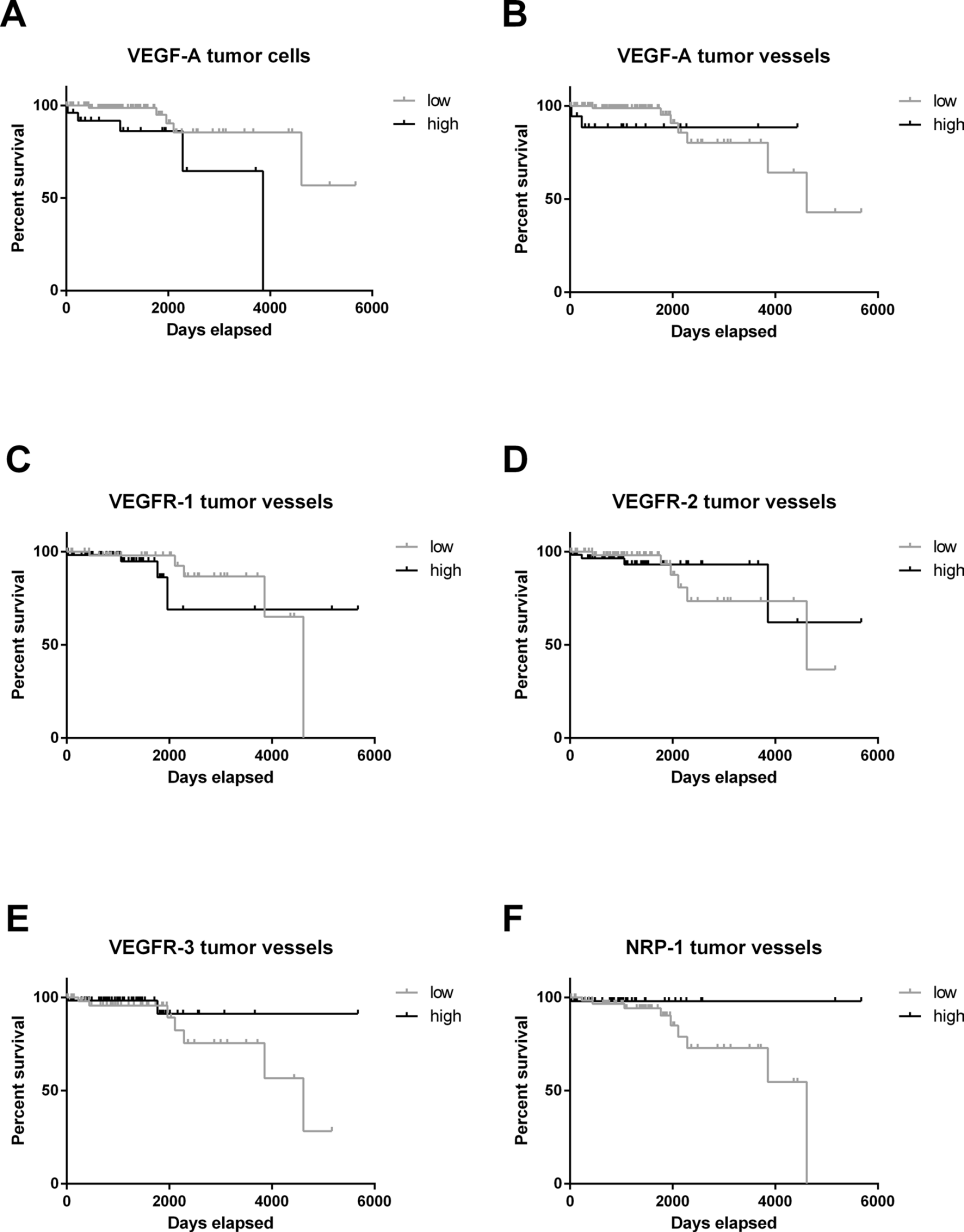
Kaplan Meier curves comparing overall survival of patients with high or low protein expression. Patients were stratified by the median. **a** VEGF-A on tumor cells was negatively correlated with patient survival (Log-rank, p = 0.0084; Wilcoxon, p = 0.0120). No significant difference was shown for (**b**) VEGF-A on tumor vessels, (**c**) VEGFR-1, (**d**) VEGFR-2, (**e**) VEGFR-3 and (**f**) NRP-1. Abbreviations: vascular endothelial growth factor (VEGF), VEGF-receptor (VEGFR), neuropilin (NRP)

VEGF-A expression level on tumor cells was negatively correlated with patient survival (median survival was undefined for low levels, and 3857 (10.6 years) for high levels, Log-rank p = 0.0084; 95% confidence interval of ratio: 0.0225 to 0.5562 and 1.798 to 44.46; Fig. 5a). All other examined factors did not influence OAS (Fig. 5b–f).

## Discussion

In our study, we investigated VEGF-A, the receptors VEGFR-1-3 and their major co-receptors NRP-1 and NRP-2 in a cohort of 147 WHO grade II and III meningioma patients in order to elaborate potential therapeutic targets. Further, we aimed to elucidate a rationale for the limited effect of former therapeutic attempts addressing angiogenesis in patients with recurrent malignant meningiomas.

We found a significant upregulation of the ligand VEGF-A in WHO grade III meningiomas compared to WHO grade II tumors. Since WHO grade III meningiomas are more likely to show necrosis, this finding may be explained by the hypoxia-driven upregulation of VEGF-A via hypoxia inducible factor (HIF)-1alpha (Shweiki et al. 1992; Ryan et al. 1998). Such observations were described in smaller populations (Reszec et al. 2015). However, we could not find significant changes in receptor- and co-receptor protein levels in WHO grade III meningiomas. This finding may also explain the lack of an upregulation of VEGF-A on tumor vessels. However, VEGF-A expression (levels) on tumor vessels may only be considered as an exploratory finding in our setting. Together with findings of an upregulation of VEGF-A and VEGFR-2 in angiomatous meningiomas (WHO I) (Nassehi et al. 2013), our data cast further doubt on a potential association of angiogenesis and malignancy in meningiomas.

To our knowledge, we are the first group reporting on NRP-1 and NRP-2 protein levels in WHO grade II and III meningiomas. We demonstrated that NRP-1 is present in a distinct proportion of tumors without significant changes in higher malignancy and without influence on PFS or OAS. We could not confirm a downregulation that has been described in angiomatous meningiomas on a mRNA level (Nassehi et al. 2013). As we already showed in glioblastomas (Baumgarten et al. 2015), NRP-2 seems to be of minor importance in meningiomas as well.

Interestingly, high protein levels of VEGFR-2 were associated with improved PFS in our cohort. This finding is in line with the findings of the phase II trial administering sunitinib and everolimus to recurring malignant meningiomas (Kaley et al. 2015). In this study, patients receiving sunitinib showed favorable PFS. In correlation with our results, this effect may be attributed to either a beneficial effect of a high VEGFR-2 expression itself, or a particularly good response of VEGFR-2-expressing tumors to sunitinib.

Our data contradict previous observations of higher VEGFR-2 levels in higher grade meningiomas and the reported association with shorter PFS (Nakada et al. 2019). This finding may be attributed to a larger cohort of WHO grade II and III meningiomas in our study. There is a lack of data concerning VEGFR-3 and the co-receptor NRP-1 that might present escape mechanisms to sunitinib treatment. Moreover, alternative angiogenetic pathways acting via angiopoietins that are upregulated in glioblastomas receiving anti-VEGF-treatment in animal models in vivo (Scholz et al. 2015) may present further promising targets and possibilities for treatment escape mechanisms in meningiomas.

Regarding recurrent disease, Preusser et al. focused their analysis on VEGF-A and only the two receptors VEGFR-1 and -2 (Preusser et al. 2012). We were not able to confirm any upregulation of VEGF-A or its receptors in recurrent tumors. Our findings show a highly significant positive correlation between VEGFR-1 and VEGF-A on both tumor cells and tumor vessels. This observation may be explained by the hypoxia-dependent regulation, not only of VEGF (Shweiki et al. 1992; Ryan et al. 1998), but also of VEGFR-1 (Gerber et al. 1997). The different expression pattern of VEGFR-2 is most likely explained by the different regulation of this receptor, as it is known to be independent of hypoxia (Ulyatt et al. 2011). The reason why VEGFR-3 is correlated with VEGF-A on tumor vessels but not on tumor cells is beyond the scope of our current study and should be investigated and discussed in further, dedicated studies.

Future targeted therapies of high-grade meningioma may include NRP-1-specific treatment, as experimentally shown by Tirand et al. (Tirand et al. 2006), who could photosensitize endothelial cells with a NRP-1 specific protein bound to the photosensitizer leading to a 25-fold increased uptake of the agent into endothelial cells. At this point however, it remains a matter of speculation if patients may benefit from such treatment. So far it has not been shown that these results are transferable to the *in vivo* situation in meningioma patients. The ongoing and actively recruiting trial NCT02847559 evaluates the effect of bevacizumab in combination with tumor-treating fields in recurrent higher WHO grade meningiomas. This may lead to new perspectives of personalized treatment strategies for these patients. Nevertheless, our data suggest that the lack of specific targets in patient sub-cohorts may mask this positive effect.

## Conclusion

The VEGF-A-driven system of tumor angiogenesis is still a target for adjuvant therapy in malignant recurrent meningioma disease. However, the role in malignant tumor progression may not be as crucial as expected. We could not show a negative influence of the VEGF-A-driven system on PFS in meningioma patients. In contrast, we identified a positive influence of higher VEGFR-2 levels associated with prolonged PFS in meningioma patients. The value of testing of the VEGF-A-driven system including its ligand and the proposed receptors prior to the administration of anti-angiogenic therapy in order to select patients for anti-VEGF, anti-VEGFR, or anti-NRP1 treatment should be evaluated in clinical trials.

## Data Availability

All data and slides are still available, please contact corresponding author.
